# Maternal linoleic acid-rich diet ameliorates bilirubin neurotoxicity in offspring mice

**DOI:** 10.1038/s41420-024-02099-9

**Published:** 2024-07-19

**Authors:** Ding Yan, XinTian Wu, Xi Chen, Jiangtuan Wang, Feifei Ge, Meixuan Wu, Jiawen Wu, Na Zhang, Min Xiao, Xueheng Wu, Qian Xue, Xiaofen Li, Jinghong Chen, Ping Wang, Daolin Tang, Xin Wang, Xin Chen, Jinbao Liu

**Affiliations:** 1 Guangzhou Municipal and Guangdong Provincial Key Laboratory of Protein Modification and Degradation, State Key Laboratory of Respiratory Disease, School of Basic Medical Sciences, The Sixth Affiliated Hospital of Guangzhou Medical University, Qingyuan People’s Hospital, Qingyuan, 511518 China; 2 Central Laboratory, the Second Affiliated Hospital of Guangzhou Medical University, Guangzhou, Guangdong 510260 China; 3 Department of Neonatology, Guangzhou Women and Children’s Medical Center, Guangzhou Medical University, Guangzhou, Guangdong 511436 China; 4 Department of Surgery, UT Southwestern Medical Center, Dallas, TX 75390 USA

**Keywords:** Neurological disorders, Cell death in the nervous system

## Abstract

Hyperbilirubinaemia is a prevalent condition during the neonatal period, and if not promptly and effectively managed, it can lead to severe bilirubin-induced neurotoxicity. Sunflower seeds are a nutrient-rich food source, particularly abundant in linoleic acid. Here, we provide compelling evidence that lactating maternal mice fed a sunflower seed diet experience enhanced neurological outcomes and increased survival rates in hyperbilirubinemic offspring. We assessed histomorphological indices, including cerebellar Nissl staining, and Calbindin staining, and hippocampal hematoxylin and eosin staining. Furthermore, we observed the transmission of linoleic acid, enriched in sunflower seeds, to offspring through lactation. The oral administration of linoleic acid-rich sunflower seed oil by lactating mothers significantly prolonged the survival time of hyperbilirubinemic offspring mice. Mechanistically, linoleic acid counteracts the bilirubin-induced accumulation of ubiquitinated proteins and neuronal cell death by activating autophagy. Collectively, these findings elucidate the novel role of a maternal linoleic acid-supplemented diet in promoting child health.

## Introduction

Bilirubin is the final metabolite of heme in mammals, primarily derived from the degradation of senescent erythrocytes [1]. The initial step in heme catabolism involves heme oxygenase (HO), which generates biliverdin, carbon monoxide (CO), and iron. Biliverdin reductase, expressed ubiquitously, converts water-soluble biliverdin into poorly water-soluble bilirubin. In the liver, bilirubin metabolism is facilitated by UDP glucuronosyltransferase family 1 member A1 (UGT1A1). This enzyme conjugates unconjugated bilirubin, increasing its water solubility and enabling excretion with bile into the intestine. The mutation in UGT1A1 results in elevated serum bilirubin, resulting in conditions like Gilbert syndrome and Crigler–Najjar syndrome [2, 3]. Uncontrolled unconjugated hyperbilirubinemia in infants can progress to acute bilirubin encephalopathy (kernicterus) and even result in fatality [4].

Recent evidence has increasingly highlighted the crucial role of bilirubin, at physiological concentrations, in modulating various biological functions, acting as a yellow hormone within the human body [5]. Bilirubin is known for its antioxidant capacity that protects the gut or other human tissues from oxidative stress [6]. However, excessive bilirubin entering the brain can cause irreversible neurological damage. The mechanisms underlying bilirubin neurotoxicity are not yet fully understood, but they may involve neuronal excitotoxicity, mitochondrial energy depletion, calcium overload, and oxidative stress [7, 8]. Our previous study revealed that the neurotoxicity of bilirubin is associated with proteasome inhibition, which is the main system for protein degradation in eukaryotic cells [9]. Exogenous bilirubin inhibits both the 19 S proteasome-associated deubiquitinases (ubiquitin-specific protease 14 [USP14] and ubiquitin carboxy-terminal hydrolase L5 [UCHL5]) and the chymotrypsin-like (CT-like) peptidase activity of 20 S proteasomes, leading to the accumulation of ubiquitinated proteins and cytotoxicity. However, effective methods to alleviate the cytotoxicity resulting from bilirubin-induced neurotoxicity through proteasome inhibition are still lacking.

It is well-known that hyperbilirubinemic *Ugt1*^−^^*/−*^ mice experiences fatal outcomes within 8 days after birth [10]. In this study, we made an incidental observation during the breeding of *Ugt1*^*+/*^^−^ female mice, which revealed a prolonged survival of hyperbilirubinemic *Ugt1*^−^^*/−*^ offspring when their maternal diet was supplemented with sunflower seeds. Our investigation not only explored the effects of adding linoleic acid-rich sunflower seeds or sunflower oil to the maternal diet on bilirubin-induced neurotoxicity in mice but also delved into the potential molecular mechanisms underlying the impact of dietary linoleic acid on bilirubin-induced neurotoxicity. This study could offer new insights into the prevention or treatment of bilirubin neurotoxicity.

## Results

### Maternal dietary supplementation with sunflower seeds prolongs survival of Ugt1^−^^*/−*^ offspring

**Fig. 1:**
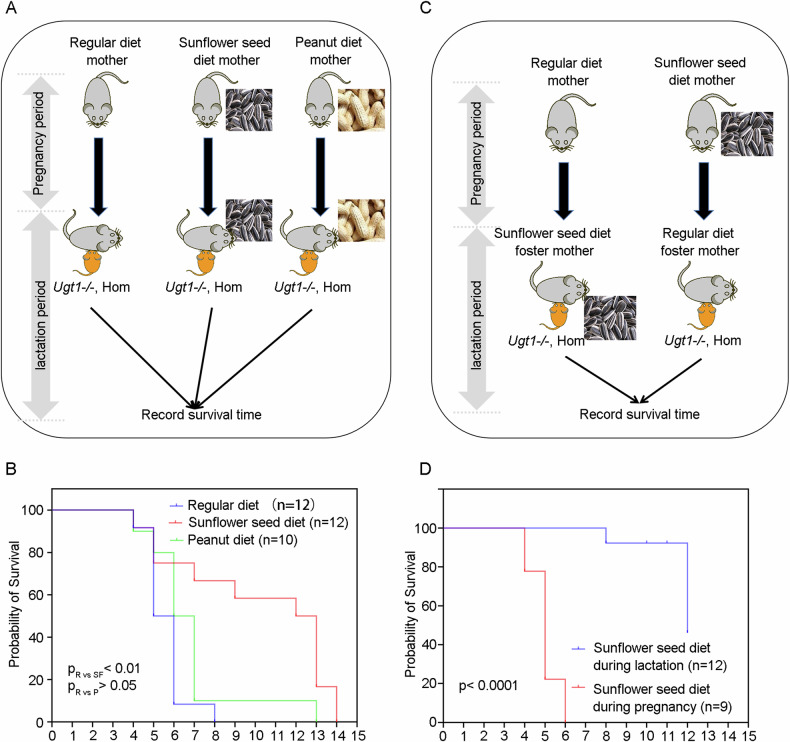
Maternal dietary supplementation with sunflower seeds prolongs survival of *Ugt1*^−^^*/−*^ offspring. **A** Schematic diagram of the groups, i.e., divided into three groups, the regular diet group, the sunflower seed diet group and the peanut diet group. **B** Survival time of hyperbilirubinemic offspring mice in the regular diet group, sunflower seed diet group and peanut diet group. Data analyses were performed using Log-rank test. **C** Schematic diagram of the groups, i.e., divided into pregnancy sunflower seed diet group and lactation sunflower seed diet group. **D** Survival time of hyperbilirubinemic offspring mice in pregnancy sunflower seed diet group and lactation sunflower seed diet group. Data analyses were performed using Log-rank test. ****p < 0.0001.

As previously described in Refs. [10, 11], *Ugt1*^−^^*/−*^ mice experience fatal outcomes within 8 days after birth due to severe hyperbilirubinemia-induced bilirubin encephalopathy, with 50% mortality at postnatal day 6 (P6). Since sunflower seeds are rich in nutrients, such as vitamin E and fatty acids, they were commonly included in the diet for breeding mice. To improve the overall breeding success of *Ugt1*^−^^*/−*^ mice, we added sunflower seeds to the cages of maternal mice [12]. Interestingly, when sunflower seeds were added to the diet of *Ugt1*^*+/*^^−^ maternal mice, we observed a prolonged survival of hyperbilirubinemic *Ugt1*^−^^*/−*^ offspring, with a median survival of postnatal 12.5 days (P12.5) (Fig. 1A, B). To investigate whether the maternal nut diet could extend the survival of hyperbilirubinemic offspring, we also added peanuts to the cages of maternal mice. However, there were no significant difference between the regular diet group and the peanut diet group (Fig. 1A, B).

Since the sunflower seed diet was administered to the mothers in the above experiments in both the pregnancy and lactation periods, the effect of the sunflower seed diet may be exerted during embryonic development, after birth, or from embryonic development to birth. To determine the specific period when the sunflower seed diet exerts its effects, we conducted a cross-foster experiment. In this study, the offspring were divided into two groups: the pregnancy sunflower seed diet group and the lactation sunflower seed diet group. In the pregnancy sunflower seed diet group, the offspring in the sunflower seed diet group were raised by mothers with a regular diet. In the lactation sunflower seed diet group, the offspring in the regular diet group were raised by mothers with the sunflower seed diet (Fig. 1C). Results showed that hyperbilirubinemic offspring in the lactation sunflower seed diet group (with a median survival of P12) exhibited a longer survival time compared to the pregnancy sunflower seed diet group (with a median survival of P5) (Fig. 1D). Thus, maternal sunflower seed diet attenuates lethal phenotype of hyperbilirubinemic offspring mice in the postnatal period, and this effect is likely transmitted to the offspring through mother’s milk.

### Maternal dietary supplementation with sunflower seeds attenuates neurological damage in Ugt1^−^^*/−*^ offspring

**Fig. 2:**
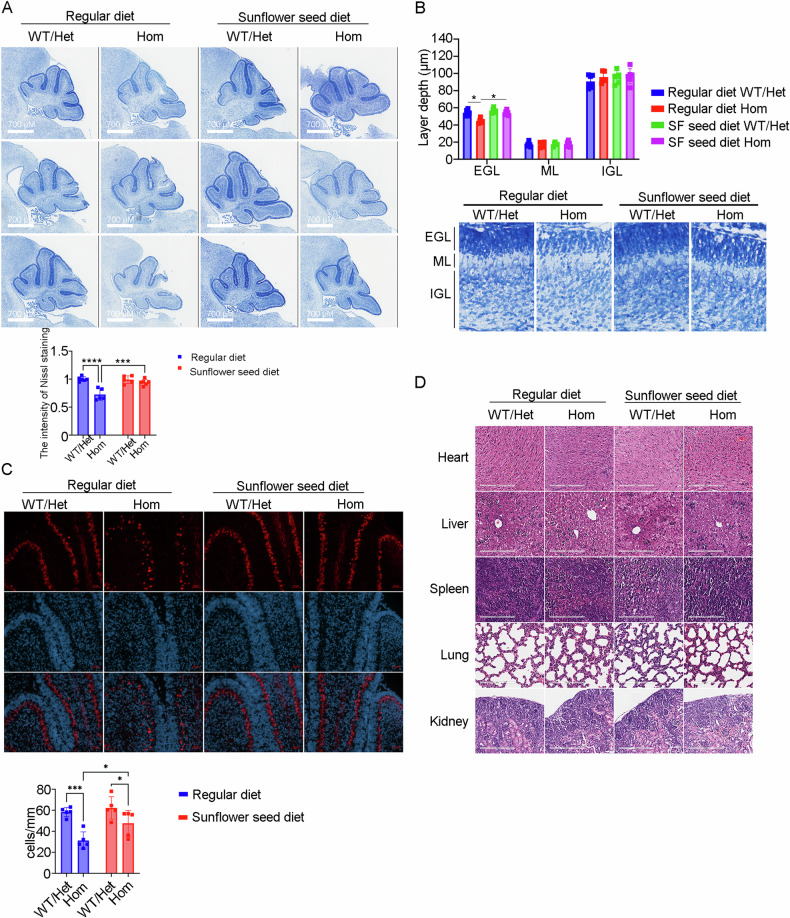
Maternal dietary supplementation with sunflower seeds attenuates neurological damage in *Ugt1*^−^^*/−*^ offspring. **A** Cerebellar Nissl staining was performed on each group of offspring mice at 5 days of age, and three representative images were presented (5-6 mice/group, 2 sections/animal). The intensities of Nissl staining were analyzed by Two-way ANOVA test. Mean ± SD. ***p < 0.001, ****p < 0.0001. **B** Upper section: the thickness of the EGL, ML, and IGL layers in lobes IV and IX of the cerebellum was individually measured for each group’s offspring. Two-way ANOVA, *p < 0.05. Lower section: representative images of each layer of the cerebellar lobe IX. **C** Representative fluorescent immunohistochemistry was performed to quantify the number of Purkinje cells (PCs) in each group of offspring mice at 5 days of age (5 mice/group, 2 sections/animal). PCs were specifically stained using an anti-calbindin1 antibody (red), while nuclei were counterstained with DAPI (blue). Two-way ANOVA test, ***p < 0.001, *p < 0.05. **D** Representative images of H&E staining in the heart, liver, spleen, lung and kidney from each group’s offspring mice.

Neonatal mice with hyperbilirubinemia often exhibit region-selective neurological injury, primarily affecting cerebellum, hippocampus, vestibule, and oculomotor nucleus [13, 14]. To further verify the beneficial effect of maternal sunflower seed diet on hyperbilirubinemic offspring mice, histomorphological examinations were performed at 5 days of age. As shown in Fig. 2A, hyperbilirubinemic homozygote *Ugt1*^−^^*/−*^ offspring (Hom) from maternal mice supplemented with regular diet demonstrated significantly fewer cerebellar Nissl vesicles, lighter staining, and slightly smaller cerebellar size, compared with wild type/heterozygote *Ugt1*^*+/*^^−^ mice (WT/Het), which was significantly improved by the maternal sunflower seed diet. Measurement of cerebellar lobules IV and IX [11] revealed a statistically significant improvement in the reduction of cerebellar external granule layer (EGL) thickness in hyperbilirubinemic offspring mice following the maternal sunflower seed diet (Fig. 2B). In addition, the cerebellum of hyperbilirubinemic offspring mice in the maternal sunflower seed diet group exhibited an increased number of Purkinje cells compared to the regular diet group (Fig. 2C).

As expected, the HE staining results indicated no significant pathological changes in organs such as the heart, liver, spleen, lung, and kidney in hyperbilirubinemic offspring mice when compared with WT/Het offspring mice, irrespective of the presence or absence of the maternal sunflower seed diet (Fig. 2D). Together, these results suggest that a maternal sunflower seed diet is beneficial for mitigating neurological damage in hyperbilirubinemic offspring mice.

### Maternal dietary supplementation with sunflower seeds does not affect the level of serum and brain bilirubin

**Fig. 3:**
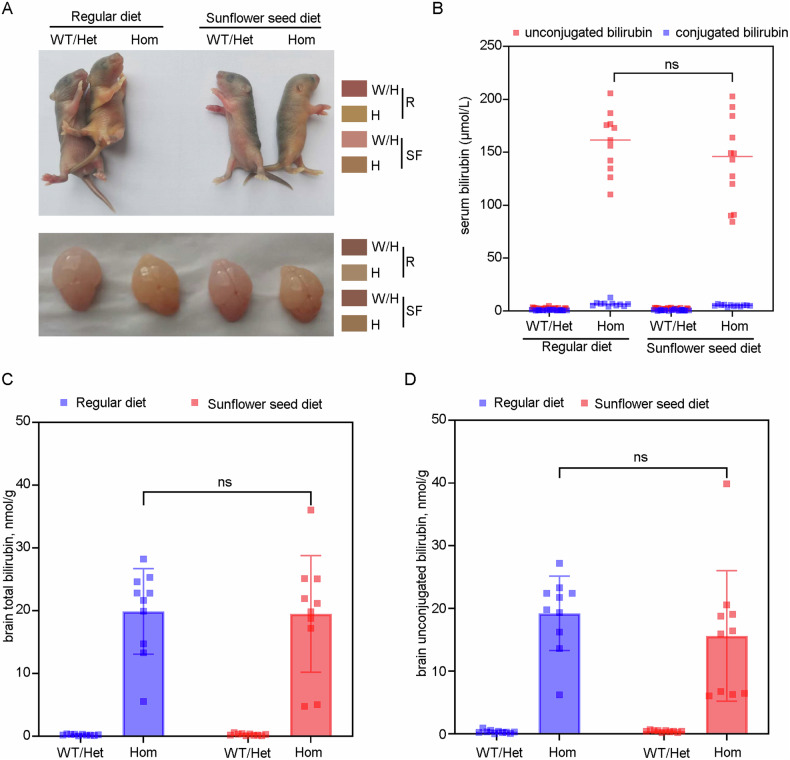
Maternal dietary supplementation with sunflower seeds does not affect the level of serum and brain bilirubin. **A** The appearance of the offspring mice and their brain tissue at 5 days of age in each group. **B**–**D** Brain and serum bilirubin levels were measured in offspring mice from each group at 5 days of age.

Next, we investigated whether maternal dietary supplementation with sunflower seeds could decrease bilirubin levels in offspring mice. We initially examined the appearance of the offspring mice and observed noticeable yellow staining in the abdomen, skin of the extremities, and brain tissue of both the regular diet group and the sunflower seed diet group (Fig. 3A). In addition, there was no statistical difference (*p* > 0.05) in serum unconjugated or conjugated bilirubin, as well as brain total or unconjugated bilirubin levels, between the two groups of hyperbilirubinemic offspring mice (Fig. 3B–D). These findings suggest that the maternal sunflower seed diet may not exert its effects by reducing serum and brain bilirubin levels in hyperbilirubinemic offspring mice.

### Maternal oral administration of linoleic acid-rich sunflower seed oil prolongs survival of Ugt1^−^^*/−*^ offspring

**Fig. 4:**
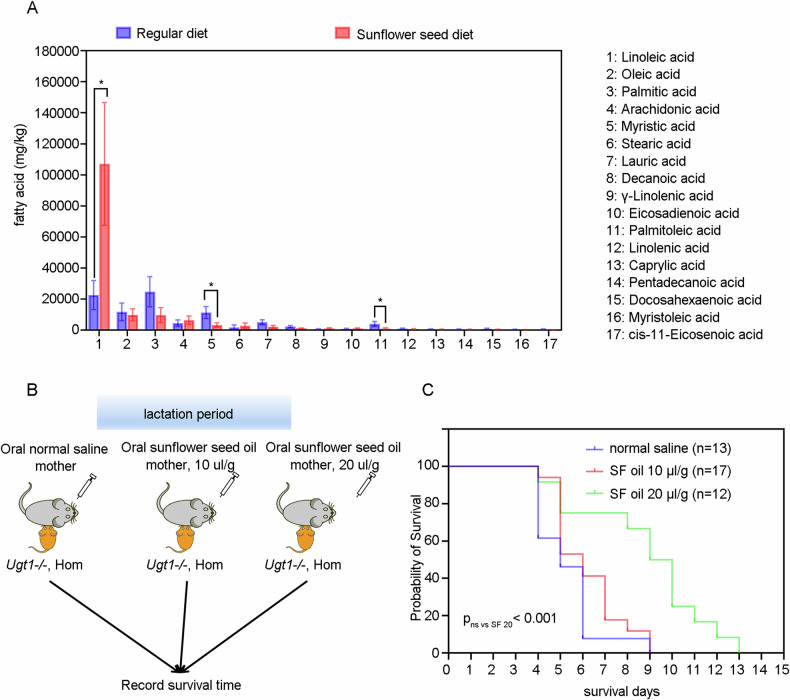
Maternal oral administration of linoleic acid-rich sunflower seed oil prolongs survival of *Ugt1*^−^^*/−*^ offspring. **A** The fatty acid components and contents in the gastric contents of offspring mice at 5 days of age were analyzed using gas chromatography-mass spectrometry. **B** Experiment schematic. **C** Survival time of hyperbilirubinemic offspring mice in normal saline group and sunflower oil group. Data analyses were performed using Log-rank test. ***p < 0.001.

Sunflower seeds contain a large amount of fat, mainly composed of unsaturated fatty acids, with linoleic acid comprising up to 70% of their content [15]. Furthermore, the fatty acid composition of milk can be influenced by maternal dietary changes among various nutrients [16]. Therefore, it is presumed that linoleic acid may play a pivotal role in the neuroprotective effects of sunflower seeds. As expected, gas chromatography-mass spectrometry analysis revealed higher levels of linoleic acid in the gastric contents of offspring mice from the sunflower seed diet group compared to the regular diet group (Fig. 4A).

To further clarify the types of effector molecules present in sunflower seeds, lactating female mice were orally administered normal saline and linoleic acid-rich sunflower oil at doses of 10 μl/g and 20 μl/g (Fig. 4B). As shown in Fig. 4C, the survival time of mice in the 20 μl/g sunflower oil group was significantly prolonged compared with the saline group, although the survival time of the 10 μl/g sunflower oil group did not exhibit a significant extension compared with the saline group. These results suggest that the predominant effector molecules in sunflower seeds are primarily present in sunflower oil, further indicating that linoleic acid may serve as the key effector molecule in sunflower seeds.

### Linoleic acid antagonizes bilirubin-induced cell death in vitro

**Fig. 5:**
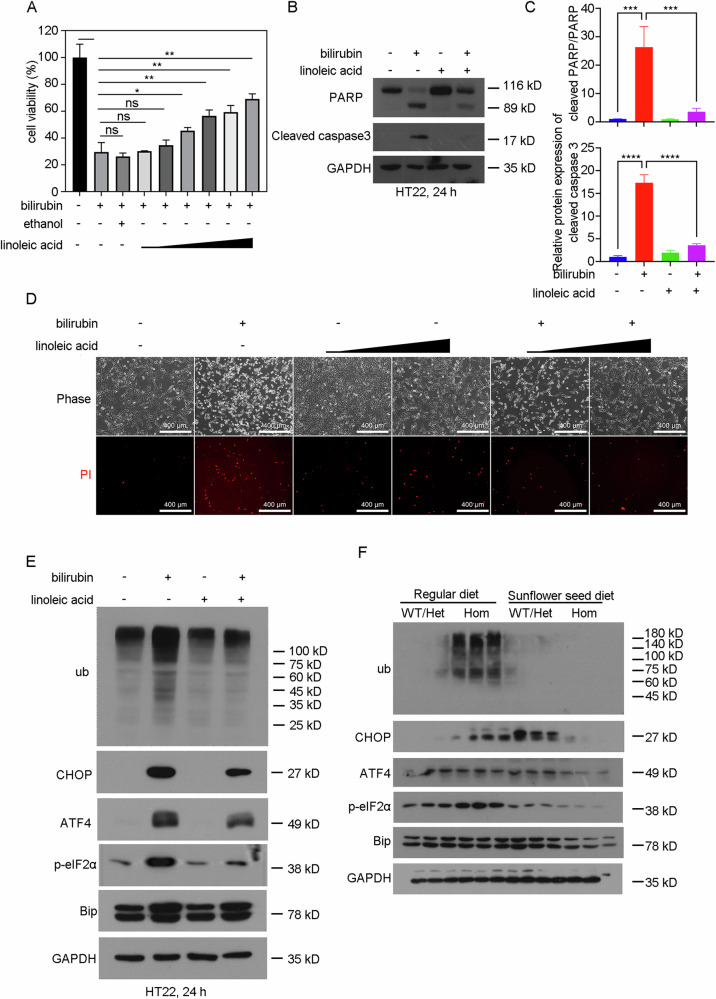
Linoleic acid antagonizes bilirubin-induced cell death in vitro. **A** Cell viability of HT22 cells treated with bilirubin (20 μM) in the presence or absence of linoleic acid (1.5625, 3.125, 6.25, 12.5, 25, 50 μg/ml) for 24 h. *p < 0.05, **p < 0.01, ***p < 0.001. **B** Western blot analysis was performed to assess the expression of the indicated protein in HT22 cells treated with bilirubin (20 μM) in the presence or absence of linoleic acid (12.5 μg/ml) for 24 h. **C** Quantification of the bands in (**B**). **D** HT22 cells were treated with bilirubin (20 μM) and/or linoleic acid (12.5 or 25 μg/ml) for 24 h, cell death was measured with Propidium iodide staining assays. **E** Western blot analysis was performed to assess the expression of the indicated protein in HT22 cells treated with bilirubin (20 μM) in the presence or absence of linoleic acid (12.5 μg/ml) for 24 h. **F** Western blot analysis was performed to assess the expression of the indicated protein in brain tissues of offspring mice at 5 days of age. The bands were quantified and normalized to GAPDH in Supplementary Fig. 1. The experiment involved three mice per group and was independently repeated three times.

To examine the effect of linoleic acid on bilirubin-induced neurotoxicity, we conducted in vitro experiments using HT22 cells, a mouse hippocampal neuronal cell line. As shown in Fig. 5A, bilirubin inhibited the cell viability of HT22, whereas linoleic acid dose-dependently ameliorated the cytotoxicity caused by bilirubin. Furthermore, bilirubin triggered the cleavage of caspase 3 and PARP, a major caspase 3 substrate, which was significantly reversed upon the addition of linoleic acid. This suggests that linoleic acid counteracts bilirubin-induced apoptosis in neuronal cells (Fig. 5B, C). Accordingly, PI staining was performed to verify that linoleic acid effectively inhibited bilirubin-induced cell death in HT22 cells (Fig. 5D and Supplementary Fig. 1A).

Previous studies have demonstrated that bilirubin induces neuronal cell death by inhibiting the ubiquitin-proteasome system and activating endoplasmic reticulum (ER) stress [9, 17, 18]. Therefore, we examined the effects of linoleic acid on bilirubin-induced ubiquitinated protein accumulation and endoplasmic reticulum stress. As illustrated in Fig. 5E and Supplementary Fig. 1B, bilirubin promoted the accumulation of ubiquitinated proteins and induced the expression of endoplasmic reticulum (ER) stress-related markers (including C/EBP homologous protein [CHOP], activating transcription factor 4 [ATF4], phosphorylation of eukaryotic translation initiation factor 2 A [p-eIF2α], and heat shock protein family A (Hsp70) member 5 [HSPA5/Bip]). These findings suggest that bilirubin inhibits ubiquitin proteasome system and induces ER stress. Consistently, linoleic acid reversed the inhibitory effects of bilirubin on the ubiquitin-proteasome system and ER stress (Fig. 5E and Supplementary Fig. 1B).

Furthermore, maternal oral administration of linoleic acid-rich sunflower seed also inhibited bilirubin-induced the accumulation of ubiquitinated proteins, CHOP, and p-eIF2α in the brain tissue of *Ugt1*^−^^*/−*^ mice (Fig. 5F and Supplementary Fig. 1C). However, neither bilirubin nor linoleic acid appeared to affect ATF4 and Bip in the brain tissue of *Ugt1*^−^^*/−*^ mice (Fig. 5F and Supplementary Fig. 1C). Collectively, these results demonstrate that linoleic acid can inhibit bilirubin-induced cell death in neuronal cells, thereby contributing to the neuroprotective effect of a sunflower seed diet against bilirubin-induced neurotoxicity.

### Linoleic acid ameliorates bilirubin-induced neurotoxicity by activating autophagy

**Fig. 6:**
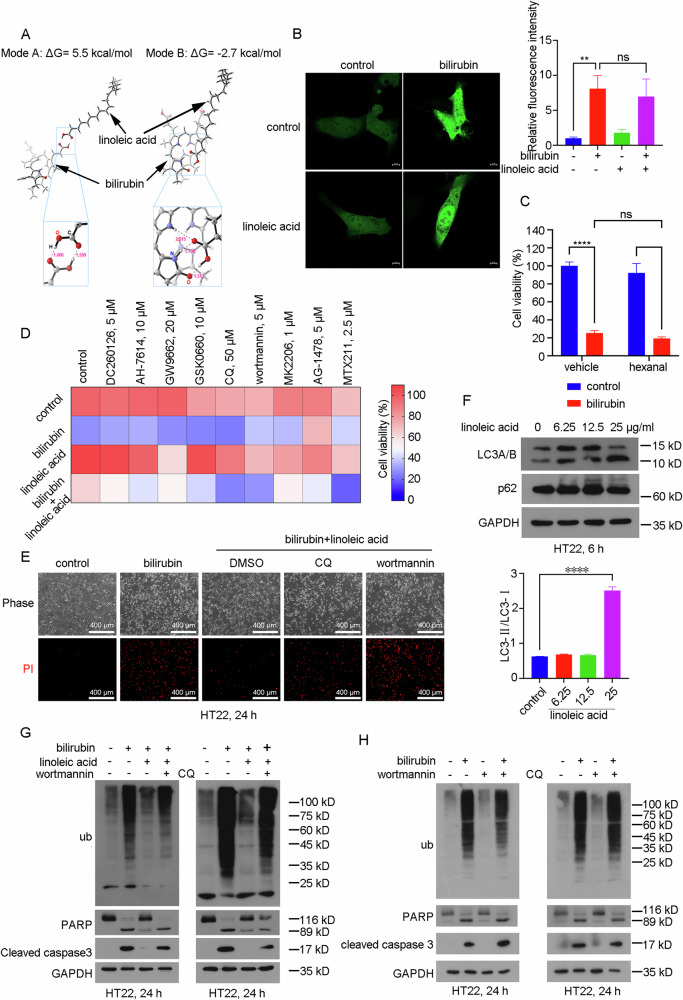
Linoleic acid may ameliorate bilirubin-induced neurotoxicity by activating autophagy. **A** Computational simulation of the interactions between bilirubin and linoleic acid in aqueous solution. **B** The HT22 cells were transfected with a plasmid encoding UnaG and subsequently exposed to bilirubin (20 μM) in the presence or absence of linoleic acid (12.5 μg/ml) for 6 hours. The samples were then subjected to fluorescence microscopy analysis. The experiment was independently repeated three times. **C** Cell viability of HT22 cells treated with bilirubin (20 μM) in the presence or absence of hexanal (12.5 μg/ml) for 24 h. **D** Cell viability of HT22 cells treated with bilirubin (20 μM) and linoleic acid (12.5 μg/ml) in the presence or absence of indicated inhibitors for 24 h. **E** HT22 cells were treated with bilirubin (20 μM) and/or linoleic acid (12.5 μg/ml) in the presence or absence of indicated inhibitors (CQ 50 μM, wortmannin 5 μM) for 24 h, cell death was measured with Propidium iodide staining assays. **F** Western blot analysis was performed to assess the expression of the indicated protein in HT22 cells treated with linoleic acid for 6 h. **G** HT22 cells were treated with bilirubin (20 μM) and/or linoleic acid (12.5 μg/ml) in the presence or absence of indicated inhibitors (CQ 50 μM or wortmannin 5 μM) for 24 h, western blot analysis was performed to assess the expression of the indicated protein. The bands were quantified and normalized to GAPDH in Supplementary Fig. 3. The experiment was independently repeated three times. **H** HT22 cells were treated with bilirubin (20 μM) in the presence or absence of indicated inhibitors (CQ 50 μM or wortmannin 5 μM) for 24 h, western blot analysis was performed to assess the expression of the indicated protein. The bands were quantified and normalized to GAPDH in Supplementary Fig. 3. The experiment was independently repeated three times.

One potential explanation is that linoleic acid might directly bind to bilirubin, thereby neutralizing its toxic effects. To investigate this possibility, we examined the binding modes of bilirubin and linoleic acid in aqueous water. Our findings revealed two likely binding modes, Mode A and Mode B (Fig. 6A). However, the binding energies of both modes A and B were low (−5.5 and −2.7 kcal/mol, respectively), indicating a weak interaction between bilirubin and linoleic acid in aqueous solution. Thus, linoleic acid may not work through direct interaction with bilirubin. UnaG is a bilirubin-dependent fluorescent protein and the binding of bilirubin to UnaG results in the emission of a fluorescence signal [19]. Interestingly, we observed that bilirubin treatment induced comparable levels of fluorescence in HT22 cells expressing UnaG protein, regardless of the presence or absence of linoleic acid (Fig. 6B and Supplementary Fig. 2A). These findings suggest that a sufficient amount of bilirubin still permeates into the cells even following linoleic acid treatment. Furthermore, hexanal, a metabolite of linoleic acid failed to attenuate bilirubin-induced cell death (Fig. 6C). We also tested the effects of other derivatives of linoleic acid, such as arachidonic acid and conjugated linoleic acid, on bilirubin-induced neurotoxicity. Interestingly, arachidonic acid exhibited a mitigating effect on bilirubin-induced cell death, whereas conjugated linoleic acid had a less pronounced effect and exhibited cytotoxicity in HT22 cells (Supplementary Fig. 2B).

Linoleic acid regulates various cell biological processes by modulating mutiple pathways or targets, including free fatty acid receptors (such as GPR40 or GPR120), PPARs, PI3K/Akt, EGFR, or autophagy [20–25]. To explore whether linoleic acid acts through these pathways or targets, we employed agents that interfere with these pathways, such as GPR40 antagonist (DC260126), GPR120 antagonist (AH-7614), PPARγ antagonist (GW9662), PPARβ/δ antagonist (GSK0660), autophagy inhibitors (chloroquine [CQ] and wortmannin), Akt inhibitor (MK-2206), EGFR inhibitor (AG 1478), and EFGR and PI3K kinase inhibitor (MTX-211). Remarkably, the protective effect of linoleic acid was substantially blocked by autophagy inhibitors (CQ and wortmannin) (Fig. 6D). Thus, we hypothesized that autophagy plays a crucial role in the neuroprotective effect of linoleic acid. Consistently, PI staining results demonstrated that linoleic acid effectively inhibited bilirubin-induced neuronal cell death, which was reversed by the addition of autophagy inhibitors (CQ and wortmannin) (Fig. 6E and Supplementary Fig. 3A).

It was reported that the activation of autophagy protects neurons and astrocytes from bilirubin-induced cytotoxicity [26]. Therefore, we hypothesized that linoleic acid antagonized the neurotoxicity of bilirubin by activating autophagy. To assess this, we examined the expression levels of LC3 and p62, which are proteins involved in the autophagy process. Our results showed that linoleic acid increased LC3-II expression but had no significant effect on p62 expression, indicating the activation of cellular autophagy by linoleic acid (Fig. 6F). Furthermore, linoleic acid attenuated bilirubin-induced the accumulation of ubiquitinated proteins and the cleavage of PARP and caspase 3. However, the protective effects of linoleic acid were blocked by the addition of autophagy inhibitors (CQ and wortmannin) (Fig. 6G and Supplementary Figure 3B), while autophagy inhibitors treatment had no effect on bilirubin-induced cytotoxicity (Fig. 6H and Supplementary Fig. 3C). Taken together, these data suggest that linoleic acid may ameliorate bilirubin neurotoxicity by activating cellular autophagy.

## Discussion

Excessive bilirubin entering the brain can cause neurotoxicity. Currently, there are some measures currently available for the prevention and treatment of bilirubin-induced neurotoxicity. Clinical methods, such as blue light therapy and exchange transfusion therapy, are commonly used to alleviate neurotoxicity associated with hyperbilirubinemia in patients with conditions like neonatal jaundice. However, both methods have certain limitations. Blue light therapy may not be effective in severe cases and can lead to adverse effects [27], while exchange transfusion requires specialized equipment and may involve procedure-related complications [28]. Thus, the development of safe, effective, and cost-efficient approaches for preventing and treating bilirubin-induced neurotoxicity is crucial.

In the present study, we investigated the effects of a maternal sunflower seed diet on the survival and neurological damage phenotype of hyperbilirubinemic *Ugt1*^−^^*/−*^ offspring mice. Our findings revealed that the maternal sunflower seed diet significantly prolonged the survival and improved the neurological damage in hyperbilirubinemic offspring mice. Additionally, we demonstrated that the effects of the linoleic acid-rich sunflower seed diet were mainly transmitted to the offspring through lactation, as shown by the cross-foster experiment. Mechanistically, linoleic acid induced the activation of autophagy, which ameliorated bilirubin-induced accumulation of ubiquitinated proteins and cell death. As sunflower seeds are a readily available and cost-effective source of linoleic acid, they hold promise for patients suffering from hyperbilirubinemia, particularly in resource-limited regions.

It has been established that unsaturated fatty acids, including linoleic acid, can readily cross the blood-brain barrier [29]. Our study further demonstrated that linoleic acid, an omega-6 polyunsaturated fatty acid, may provide neuroprotection against hyperbilirubinemia-induced neurological damage in mice. Other studies have suggested that docosahexaenoic acid (DHA), an omega-3 polyunsaturated fatty acid, exerts neuroprotective effects against bilirubin-induced neurotoxicity by enhancing the activity of superoxide dismutase (SOD) and catalase (CAT) [30, 31]. It is worth noting that under normal physiological conditions, unsaturated fatty acids may compete with bilirubin for binding sites on albumin in the bloodstream, potentially leading to increased levels of free bilirubin [32]. Moreovers, high doses of unsaturated fatty acids (including linoleic acid) can act as inhibitors of UGT1A1 in vitro, potentially hindering the conversion of unconjugated bilirubin into conjugated bilirubin and resulting in elevated levels of unconjugated bilirubin [33]. These properties of unsaturated fatty acids suggest a complex role in the management of hyperbilirubinemia.

Our previous study demonstrated that bilirubin can induce neurotoxicity by inhibiting the proteasome, leading to the accumulation of ubiquitinated proteins [9]. In the present study, we discovered that linoleic acid, abundant in sunflower seeds, significantly mitigates bilirubin-induced ubiquitinated protein accumulation both in vitro and in vivo. Apart from its neurotoxic effects, bilirubin is a potent antioxidant that plays crucial physiological roles in the human body. Therefore, instead of simply reducing serum bilirubin levels, linoleic acid may preserve other properties of bilirubin, such as its antioxidant activity, while counteracting its neurotoxicity. Additionally, these effects of linoleic acid appear to be attributed to the activation of autophagy. Hence, we propose that linoleic acid may counteract bilirubin-induced neurotoxicity by inducing autophagy. It has been reported that linoleic acid activates autophagy through both mTOR-dependent and independent pathways [20]. Future studies should delve into the detailed mechanisms of linoleic acid-induced autophagy activation.

However, the present study has certain limitations. Firstly, sunflower seeds were not the exclusive dietary source for the maternal mice in the sunflower seed group during the experiment, potentially introducing bias due to maternal food preferences. Secondly, although we compared whole-brain bilirubin levels, cerebellar bilirubin levels were not assessed. Furthermore, it is essential to calculate the free bilirubin as a crucial parameter to delve into the detailed mechanism of fatty acid antagonism against bilirubin-induced cytotoxicity. Thirdly, while the combination of phototherapy and sunflower seed diets may hold promise as a method for preventing and treating hyperbilirubinemia, there is presently insufficient experimental evidence to firmly support this claim.

**Fig. 7:**
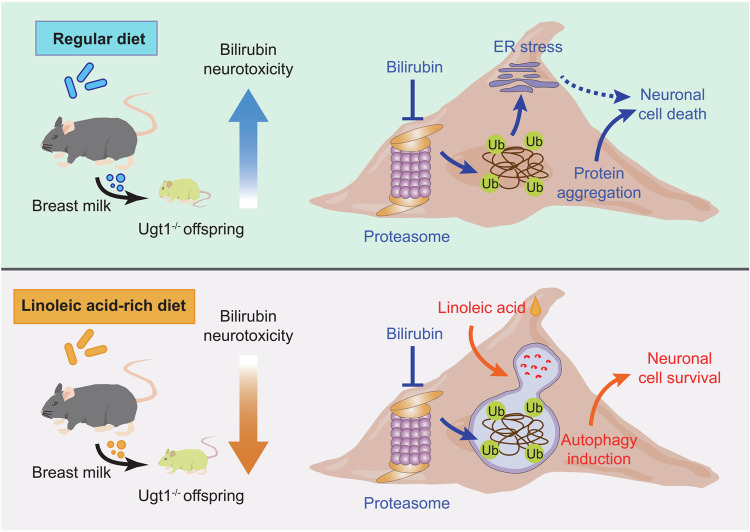
A working model of maternal linoleic acid-rich diet in hyperbilirubinemic offspring. During lactation, a maternal linoleic acid-rich diet increases the linoleic acid content in milk, which is then transmitted to hyperbilirubinemic offspring mice through breastfeeding. Mechanistically, the activation of autophagy by linoleic acid antagonizes the neurotoxic effects of bilirubin.

In conclusion, our study provides novel insights into the protective effect of a maternal sunflower seed diet on hyperbilirubinemic offspring mice, achieved through breastfeeding during lactation. Furthermore, we propose that this protective effect of sunflower seeds can be attributed to linoleic acid, which alleviates bilirubin-induced accumulation of ubiquitinated proteins and neuronal cell death by activating autophagy (Fig. 7). These findings highlight the potential of dietary interventions involving linoleic acid-rich sources, such as sunflower seeds, as a promising strategy for preventing and treating bilirubin-induced neurotoxicity.

## Materials and methods

### Reagents

Bilirubin (#B4126) was purchased from Merck. Linoleic acid (#90150) was purchased from Cayman. DC260126 (#HY-101906), AH-7614 (#HY-19996), GW9662 (#HY-16578), GSK0660 (#HY-12377), chloroquine (#HY-17589A), Wortmannin (#HY-10197), MK2206 (#HY-10358), AG-1478 (#HY-13524), and MTX-211 (#HY-107364) were purchased from MedChemExpress. PARP (#9532), cleaved caspase 3 (#9661), CHOP (#2895), ATF4 (#11815), p-eIF2α (#3398), Bip (#3177), and LC3A/B (#4108) antibodies were purchased from Cell Signaling Technology. Ubiquitin antibody (#sc-8017, 1:500) was purchased from Santa Cruz biotechnology. P62 (#ab56416) was purchased from Abcam. GAPDH (#AP0063) was purchased from Bioworld. With the exception of the specified antibodies, all other antibodies utilized for western blot experiments were diluted at a ratio of 1:1000.

### Cell lines

HT22 mouse hippocampal neuronal cells were obtained from Wuhan Procell Life Science & Technology Company (CL-0697) and cultured in Dulbecco’s modified Eagle medium (DMEM) supplemented with 10% fetal bovine serum. The cells were recently authenticated, tested for mycoplasma contamination, and maintained at 37 °C in a humidified atmosphere with 5% CO_2_.

### Animals

The study was conducted according to the guidelines of the Declaration of Helsinki. All animal handling procedures were performed in compliance with the People’s Republic of China legislation for the care and use of laboratory animals. The experimental protocols involving animals were approved by the Institutional Animal Care and Use Committee of Guangzhou Medical University. Heterozygous Ugt1 knockout adult male mice (Het; *Ugt1*^*+/*^^−^) in the C57BL/6 background were generously provided by Professor Zhongqiu Liu from Guangzhou University of Chinese Medicine [10, 34]. Homozygous Ugt1 knockout mice (Hom; *Ugt1*^−^^*/−*^) were generated by breeding *Ugt1*^*+/*^^−^ mice. The parental mice were housed in the Animal Center of Guangzhou Medical University, following a 12 h light/12 h dark circadian rhythm, with free access to water. The mice were randomly allocated to either control or treatment group. Both control and treatment groups of parental mice were fed with regular chow diet, while the treatment group received additional autoclaved shelled sunflower seeds or peanuts in their cages alongside the regular chow diets. The regular chow diets for the parental mice were provided by Guangdong Medical Laboratory Animal Center.

For the assessment of orally administered sunflower oil, autoclaved pure sunflower oil (without being dissolved in other solvents) was administered to lactating female mice via gavage at the indicated dose (10 ul/g or 20 ul/g once daily), commencing on the first day of pup’s life. Throughout the gavage period, females had ad libitum access to water and a regular diet. The assay continued until all hyperbilirubinemic offspring mice succumbed.

### Cerebellar histological detection

The brains were carefully dissected from the skulls and fixed in 4% paraformaldehyde for 24 h. After fixation, the brains were dehydrated by immersion in a 20% sucrose solution, followed by transfer to a 30% sucrose solution until they settled. Excess sucrose solution was removed by gently blotting with filter paper. Prior to sectioning, the cryostat and sample base were pre-cooled. The brains were then bisected along the median sagittal plane using a blade. To ensure optimal orientation, the brain sections were meticulously embedded layer by layer with OCT embedding agent, ensuring that the largest section was parallel to the base and positioned facing upwards. The resulting sections had a thickness of 20 μm.

For the assessment of cerebellar development in neonatal offspring, Nissl staining and Calbindin staining were performed on neonatal mice at 5 days of age (*n* = 5–6 animals/group; 1–2 sections/animal).

For Nissl staining, we utilized the corresponding reagents provided by Servicebio Biotechnology and applied it to paraformaldehyde-fixed sections in accordance with the manufacturer’s instructions.

For Calbindin staining, after blocking with 3% BSA for 30 minutes, the primary antibody (abcam#ab108404, 1:150) was incubated overnight at 4 °C. Following three washes with PBS, a fluorescent secondary antibody incubation was performed and subsequently, the nuclei were re-stained using DAPI.

To capture images of the stained sections, an Aperio digital pathology slide scanner was employed.

### Measurement of serum bilirubin

After administering anesthesia to the offspring mice, a gentle inversion of the mice was performed to facilitate blood flow towards the head and face. The skin in the submandibular region was carefully disinfected using alcohol-soaked cotton balls. A small incision was then made in the submandibular vein using a blade to collect blood samples.

Microcapillary blood collection tubes were used to collect the blood, which was transferred into eppendorf tubes (EPs). The collected blood samples were incubated at room temperature in the dark for 30 min. After incubation, the tubes were subjected to centrifugation at 3000 rpm for 10 min at room temperature. This centrifugation step allowed for the separation of serum from the cellular components of the blood. The resulting serum was carefully transferred to another EP tube and stored appropriately for further analysis.

To determine the concentrations of direct and total bilirubin in the serum, a biochemical analyzer was utilized (Hitachi Ltd. 3100 Serial, Tokyo, Japan). This analyzer employs specific assays or reagents to measure the bilirubin levels accurately. The serum was diluted 2-fold prior to being tested on the biochemical analyzer. Approximately 10 μl of diluted serum is consumed per test. Additionally, the concentration of indirect bilirubin was calculated based on the total (Total BilE-HA, Wako, Japan) and direct (Direct Bil E-HA, Wako, Japan) bilirubin measurements.

### Measurement of brain bilirubin

In order to detect brain bilirubin, we have referenced a research paper and made enhancements based on it [35]. Preparation of samples for measurement of total bilirubin: The brains were extracted from the skull. After homogenizing the brain tissue, transfer 20 mg into a homogenization tube. Add exactly 200 μl of 10% oxalic acid and 200 μl of water-saturated dichloromethane. Seal the tube tightly and wrap it in tin foil to protect it from light. Shake the mixture overnight at a temperature of 4 °C using a 360 rotary shaker. Afterward, centrifuge the sample at 7000 rpm for 5 min at a temperature of 4 °C. Carefully collect the lower layer containing water-saturated dichloromethane, filter it, and use it for analysis under the corresponding chromatographic conditions.

Preparation of samples for measurement of indirect bilirubin: Follow a similar method to that used for total bilirubin, but replace the 10% oxalic acid and water-saturated dichloromethane with dichloromethane. The chromatographic analysis was performed using an ODS-2 column (250 mm × 4.6 mm, 5 μm) under the following conditions: isocratic elution with a mobile phase consisting of acetonitrile - 1% glacial acetic acid aqueous solution (95:5), at a volume flow rate of 1 ml/min. The detection wavelength was set at 450 nm, and the column temperature was maintained at 30 °C. An injection volume of 4 μl was used.

### Fatty acid determination of gastric contents

Gastric contents were collected on ice. Firstly, the entire gastric capsule was excised from cardia to pylorus, followed by careful dissection of the gastric wall with scissors. Subsequently, the gastric contents were meticulously stripped into 1.5 ml centrifuge tubes and stored at −80 °C.

For fatty acid determination of gastric contents, 2 mL of petroleum ether-ether mixture and 1 ml of potassium hydroxide-methanol solution as methylation reagent were added to 500 mg (to 0.1 mg) of homogeneous gastric in a stoppered tube with a capacity of 10 ml. The mixture was vortexed and shaken, and incubated at 65 °C for 1 h. Then, the mixture was vortexed and shaken again, and 2 ml of deionized water was added, allowing the reaction to separate for 30 min. Finally, the mixture was centrifuged at a speed of 4500 rpm for 2 min before and the supernatant were analyzed by gas chromatography-mass spectrometry (Agilent Technologies).

### Cell viability assay

HT22 cells were seeded in 96-well plates. After allowing the cells to adhere, they were treated with experimental compounds for 24 h. The reagents were dissolved in RPMI-1640 medium with 10% fetal bovine serum. After the treatment period, 10 µl of Cell Counting Kit-8 reagent (DOJINDO, CK04) was added to each well, followed by incubation for 2 h at 37 °C. The absorbance at a wavelength of 450 nm was then measured using a microplate reader (Thermo Scientific).

### Propidium iodide staining

HT22 cells were seeded in 24-well plates. After treatment with the indicated reagents in RPMI-1640 medium with 10% fetal bovine serum for 24 hours, the cells were subjected to propidium iodide (PI) staining for 30 min using the PI staining kit (KeyGEN BioTECH#KGA108). The PI-positive cells were then imaged using a fluorescence microscope (ZEISS).

### UnaG assay

The HT22 cells were cultured in glass-bottomed dishes, and after they adhered to the surface, the UnaG plasmid was transfected using Lipofectamine 3000 reagent according to the manufacturer’s instructions. After 24 hours, HT22 cells expressing UnaG were treated with 20 μM bilirubin in the presence or absence of linoleic acid. Subsequently, fluorescence microscopy was employed to observe cellular fluorescence after a 6-hour incubation period for each experimental group.

### Western blotting

Western blotting was performed following a previously described protocol [36]. Briefly, equal amounts of protein extracts were separated by SDS-PAGE and electrotransferred to a polyvinylidene difluoride (PVDF) membrane (Millipore). Subsequently, the membranes were blocked with 5% non-fat milk for 1 h at room temperature. Overnight incubation with primary antibodies was conducted at 4 °C. The following day, horseradish peroxidase-labeled secondary antibodies were applied and incubated for 1 h at room temperature. Finally, the membranes were treated with an ECL reagent and visualized using x-ray film.

### Statistical analysis

The data were analyzed using Graphpad Prism 9.0 software. Survival analysis was conducted by Log-rank test. The statistical analyses for group comparisons were conducted using t-tests and ANOVA, respectively, in accordance with the experimental design. The results are presented as mean ± standard deviation. A *p* value less than 0.05 indicates statistical significance.

### Supplementary information

Supplemental figures and figure legends

Full and uncropped western blots

## Data Availability

The data supporting the present findings are available from the corresponding author upon reasonable request.
