# Supplementary material for: Maternal linoleic acid-rich diet ameliorates bilirubin neurotoxicity in offspring mice
Source: Cell Death Discov. 2024 Jul 19;10:329. doi: 10.1038/s41420-024-02099-9 (PMC11271588; doi:10.1038/s41420-024-02099-9)
Supplement: Supplementary file 1 — Supplemental figures and figure legends [file 41420_2024_2099_MOESM1_ESM.docx]

**Supplemental figures and figure legends**


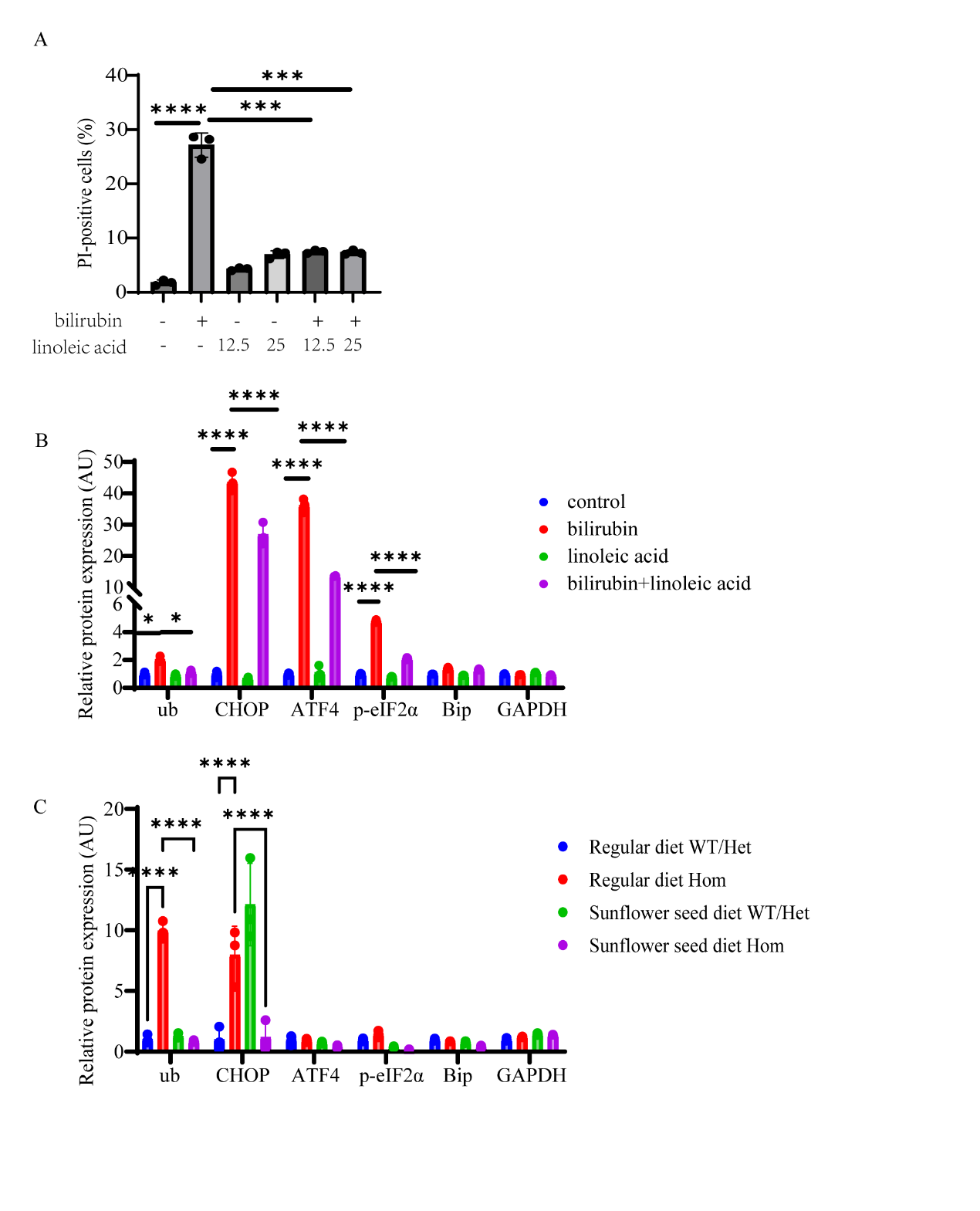


**Figure S1. Diagram for quantitative analysis of western blots in Figure 5.**

1. Quantitative analysis of PI-positive cells in Figure 5D. (B) Quantitative analysis of western blots in Figure 5E. (C) Quantitative analysis of western blots in Figure 5F. Two-way ANOVA test, ****p< 0.0001, ***p< 0.001, *p< 0.05.


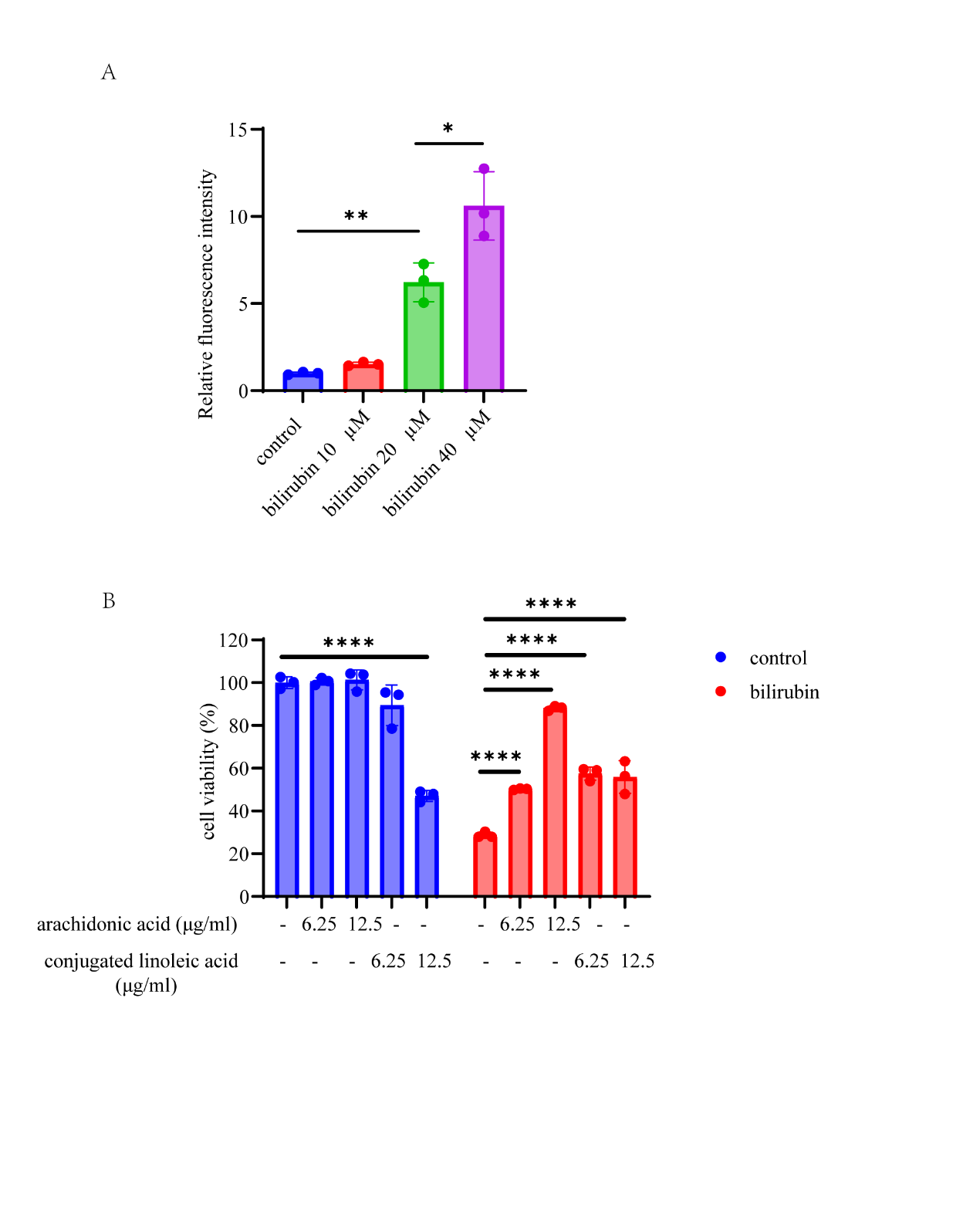


**Figure S2.** **Arachidonic acid and conjugated linoleic acid attenuate bilirubin-induced cell death.**

1. The HT22 cells were transfected with a plasmid encoding UnaG and subsequently exposed to bilirubin (10 μM, 20 μM, and 40 μM) for 6 hours. The samples were then subjected to fluorescence microscopy analysis. The experiment was independently repeated three times. (B) Cell viability of HT22 cells treated with bilirubin (20 μM) in the presence or absence of arachidonic acid or conjugated linoleic acid for 24 h. Two-way ANOVA test, ****p< 0.0001, **p< 0.01, *p< 0.05.


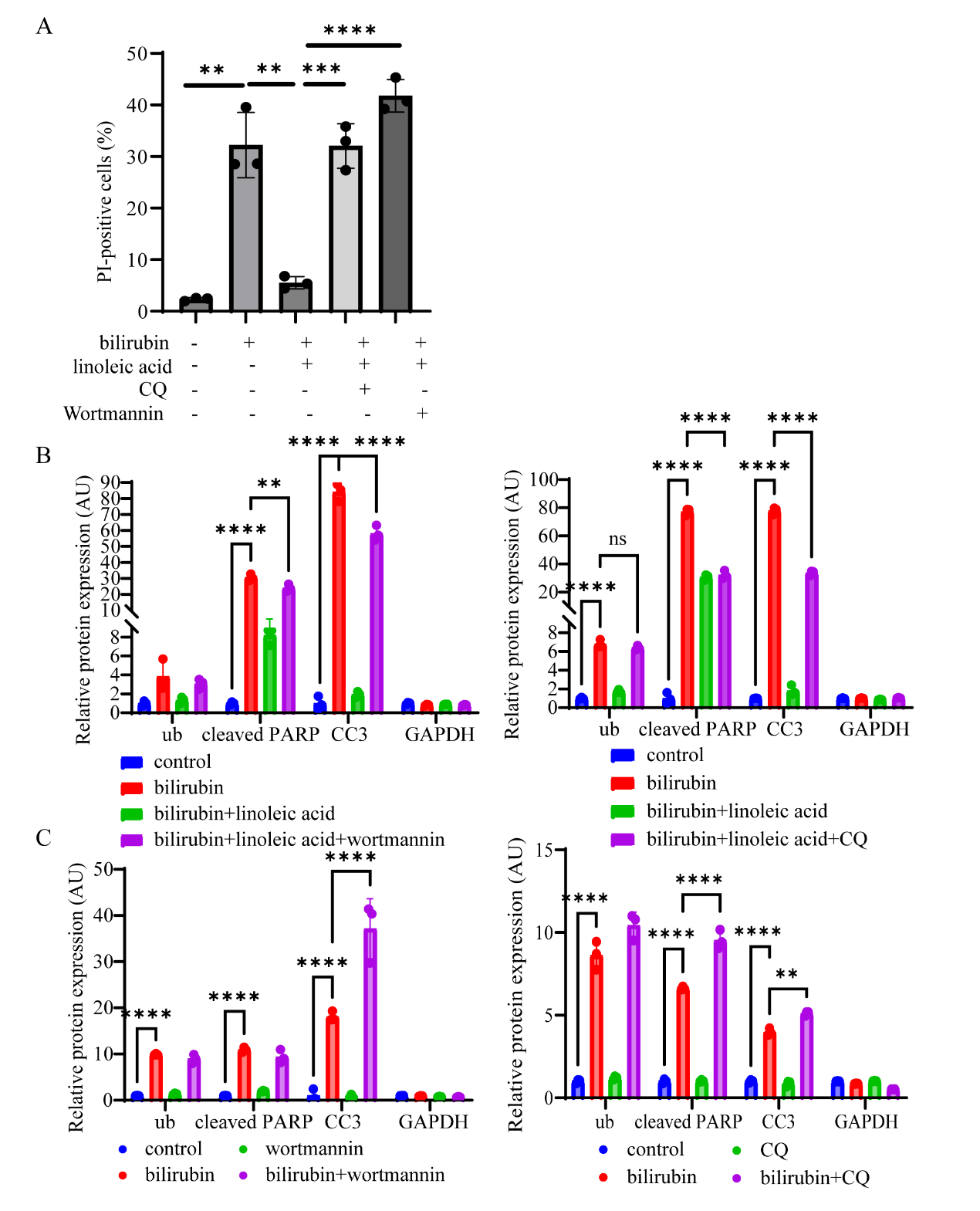
**Figure S3. Diagram for quantitative analysis of western blots in Figure 6.**

1. Quantitative analysis of PI-positive cells in Figure 6E. (B) Quantitative analysis of western blots in Figure 6G. (C) Quantitative analysis of western blots in Figure 6H. Two-way ANOVA test, ****p< 0.0001, ***p< 0.001, **p< 0.01. CC3, cleaved caspase 3.
