# Supplementary material for: Maternal linoleic acid-rich diet ameliorates bilirubin neurotoxicity in offspring mice
Source: Cell Death Discov. 2024 Jul 19;10:329. doi: 10.1038/s41420-024-02099-9 (PMC11271588; doi:10.1038/s41420-024-02099-9)

Original image: Figure 5B

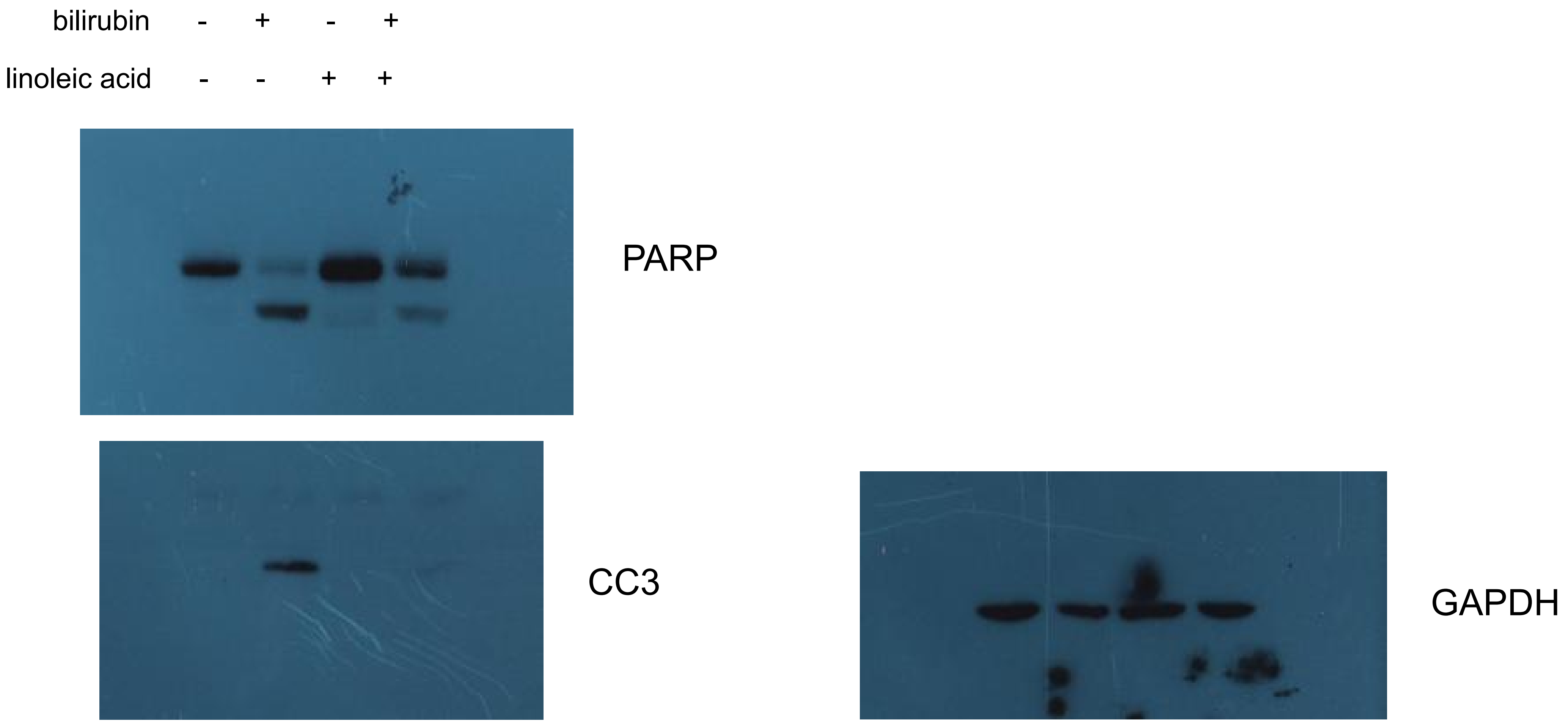

Original image: Figure 5E

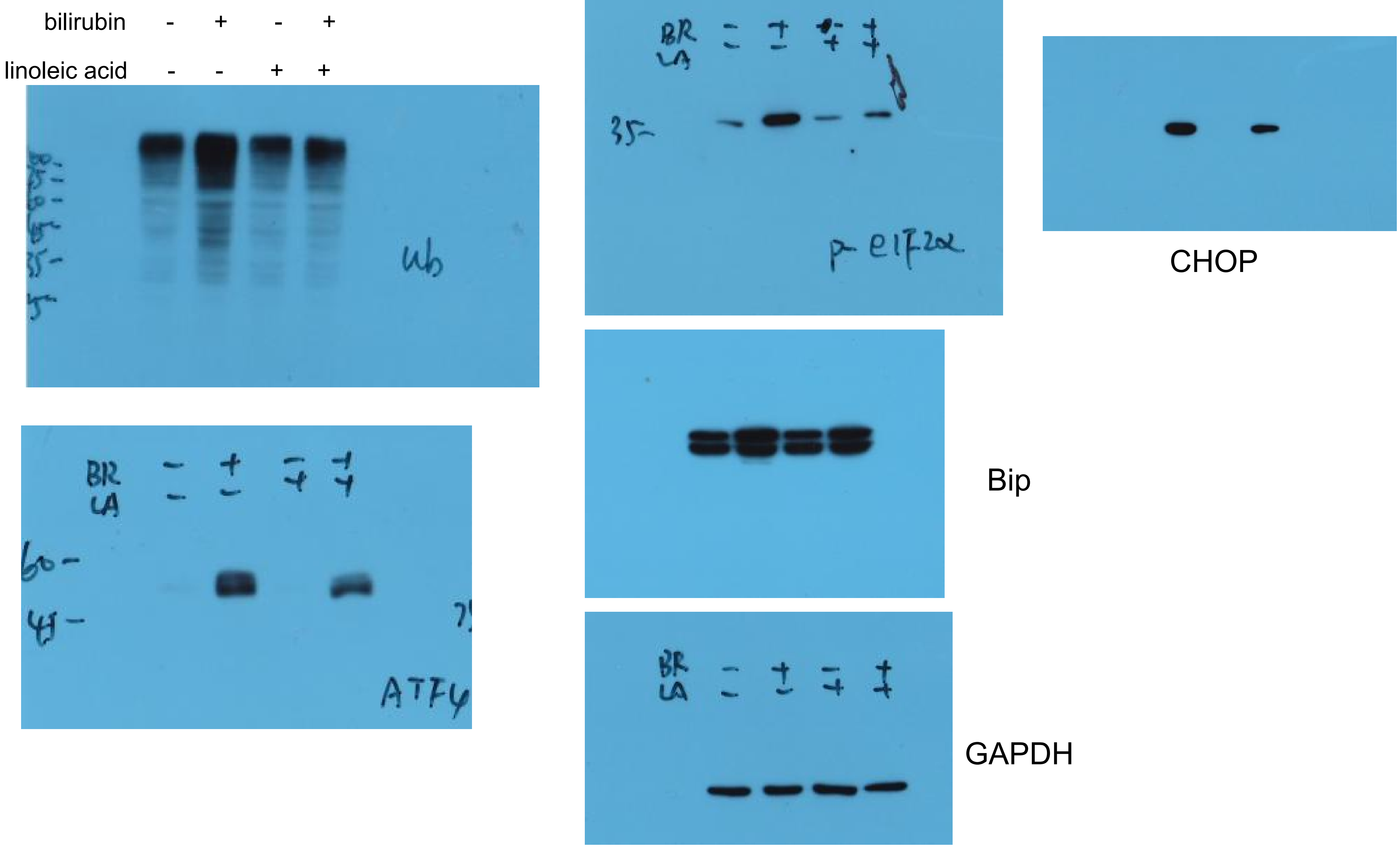

Original image: Figure 5F

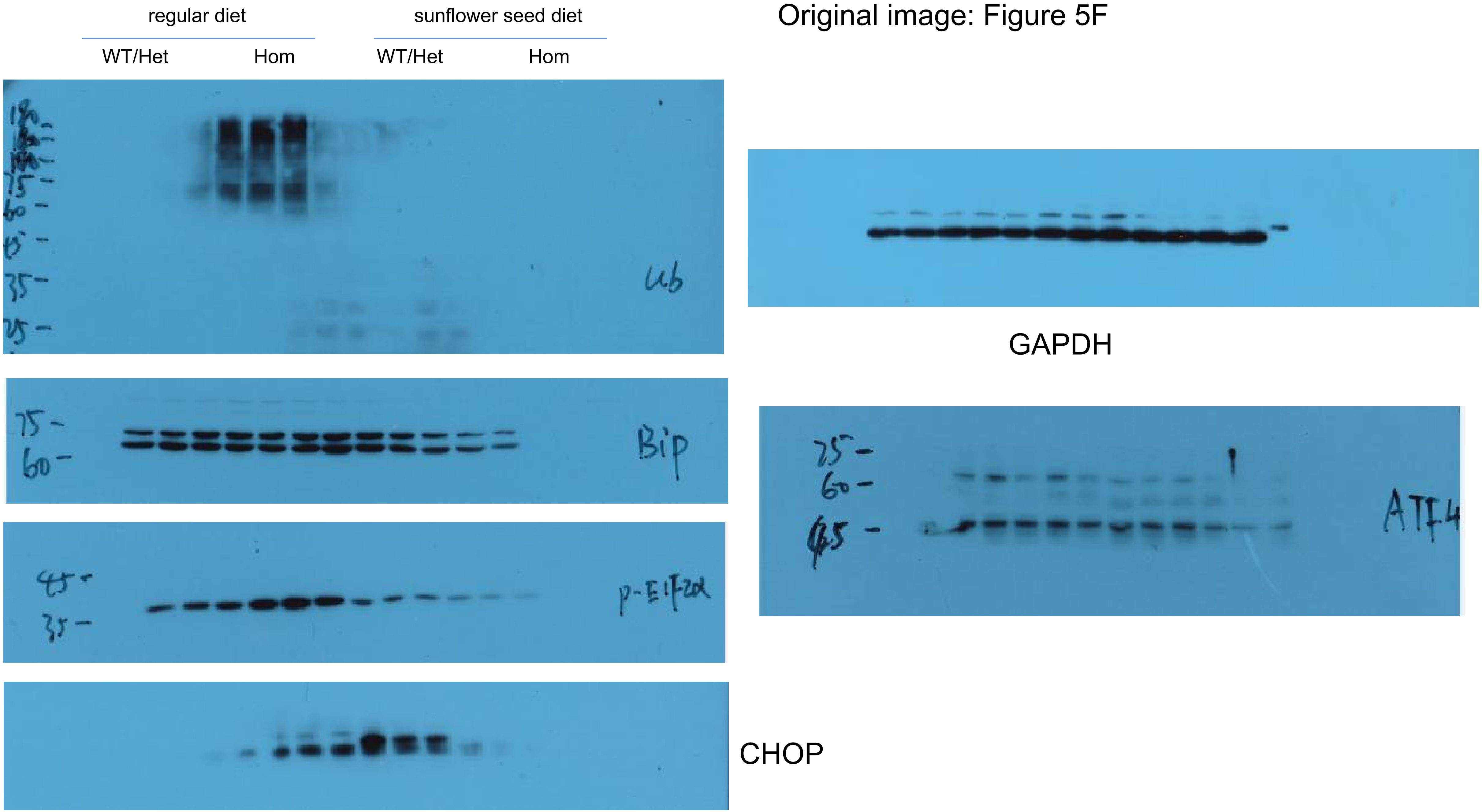

Original image: Figure 6F

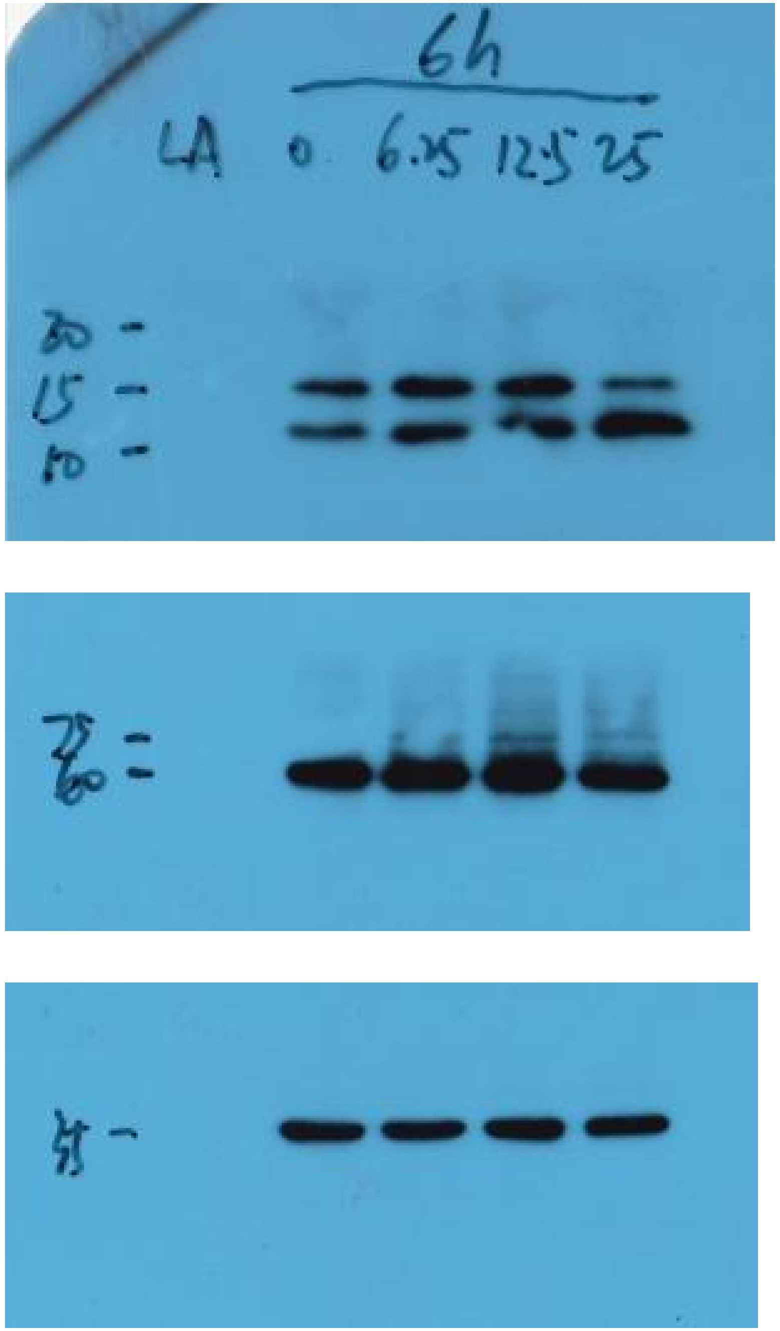

LC3/A/B

p62

GAPDH

Original image: Figure 6G

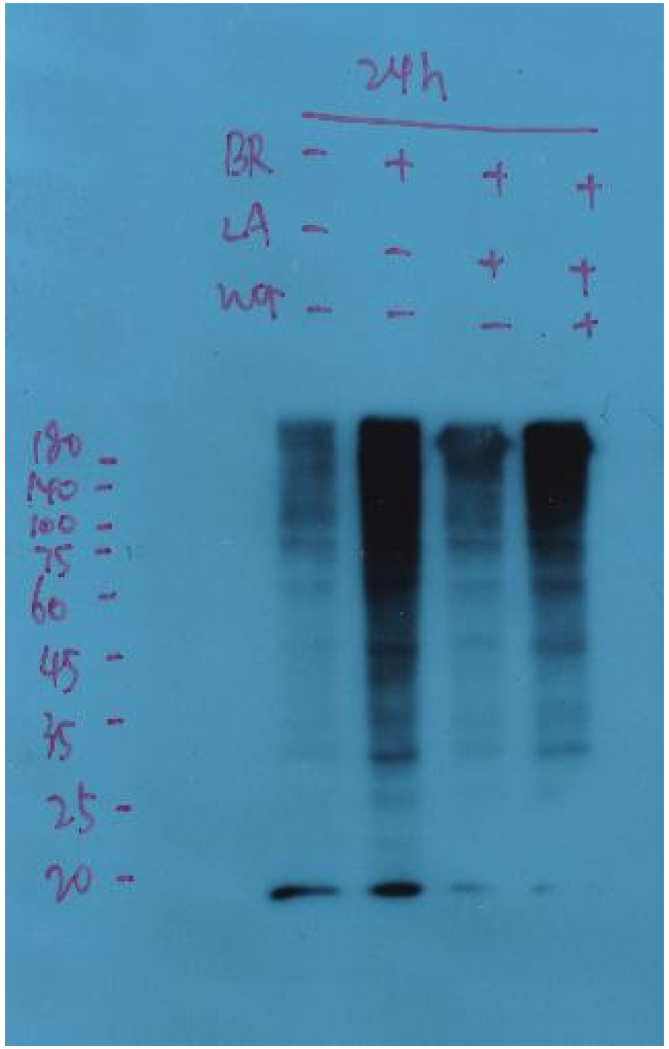

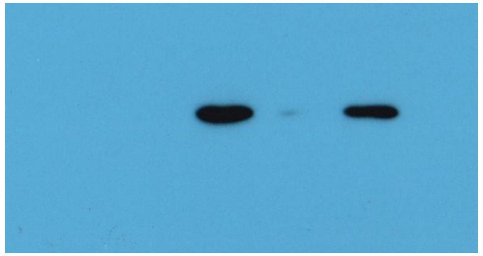

CC3

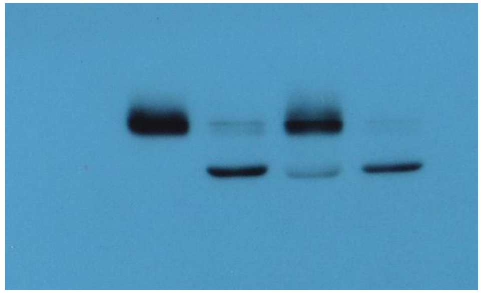

PARP

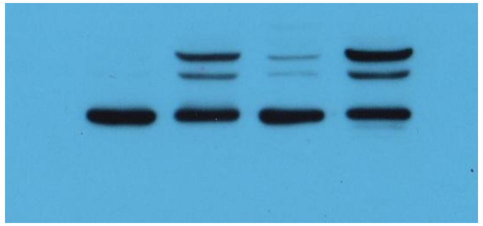

GAPDH

Original image: Figure 6G

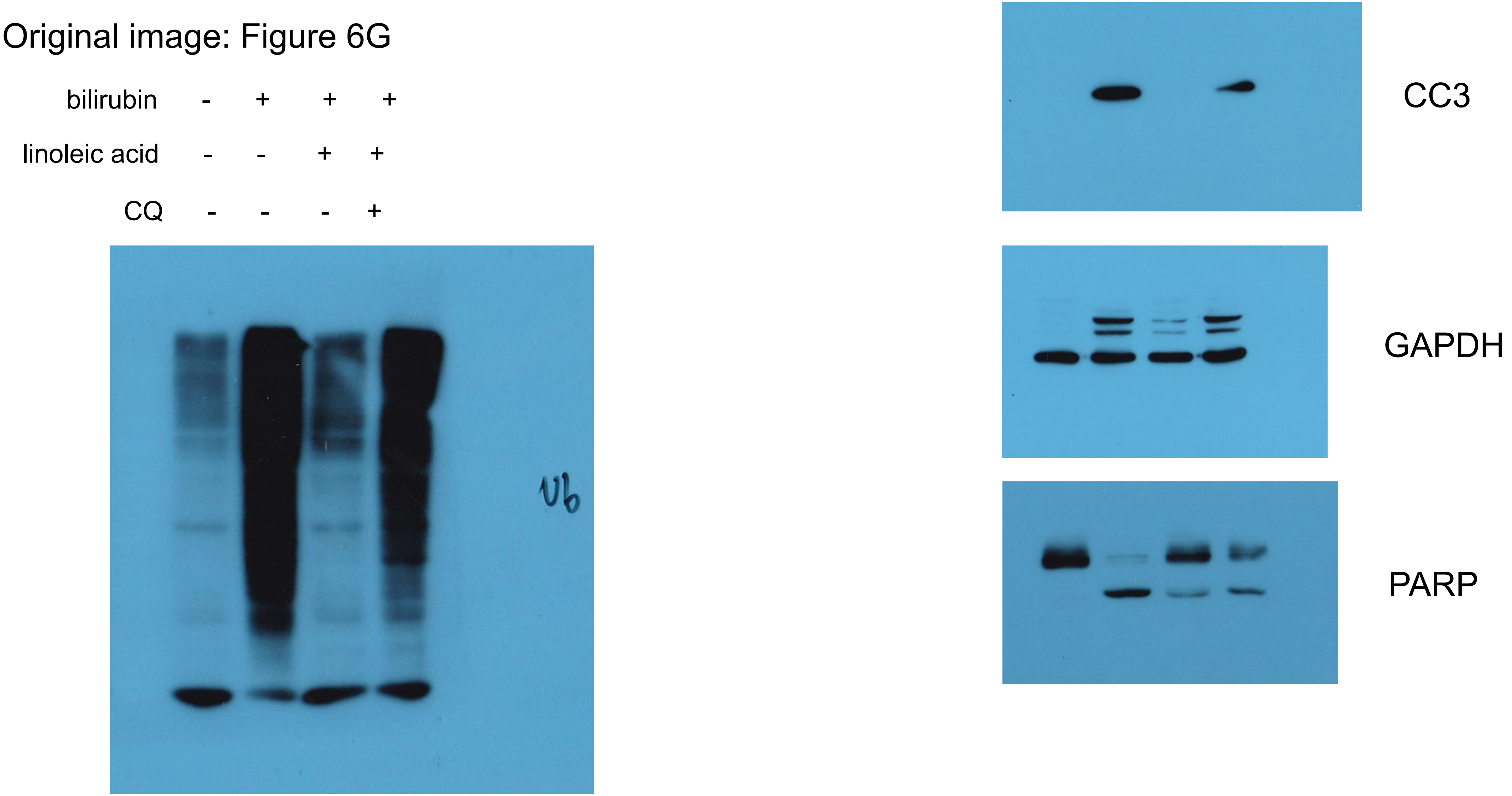

Original image: Figure 6H

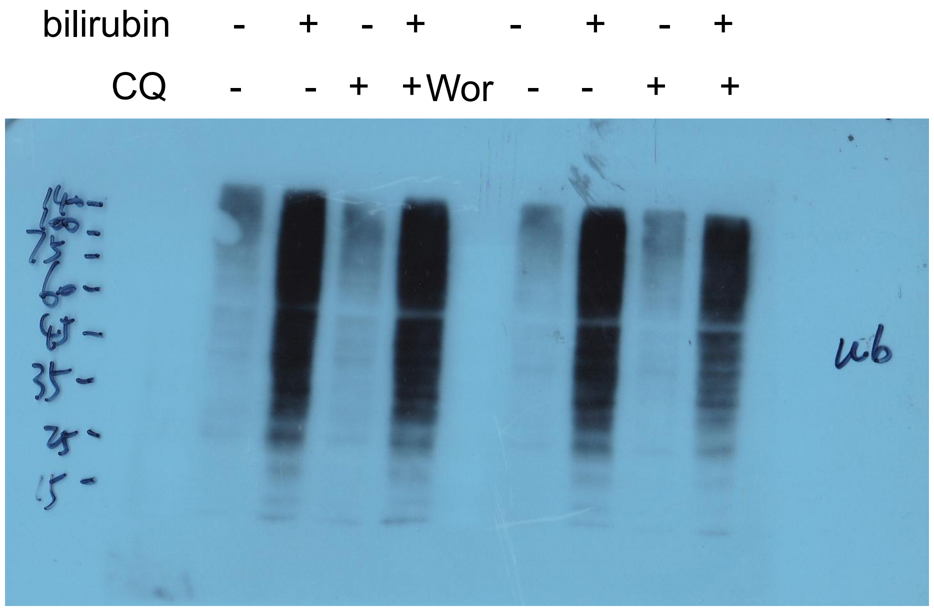

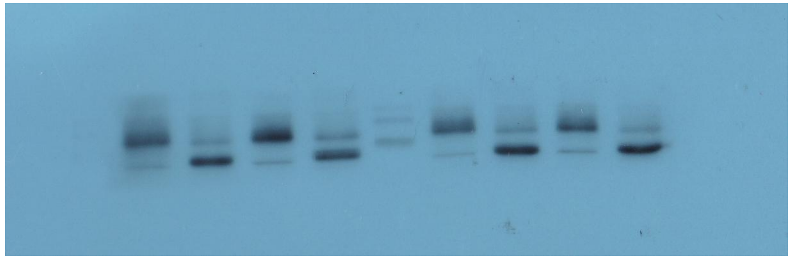

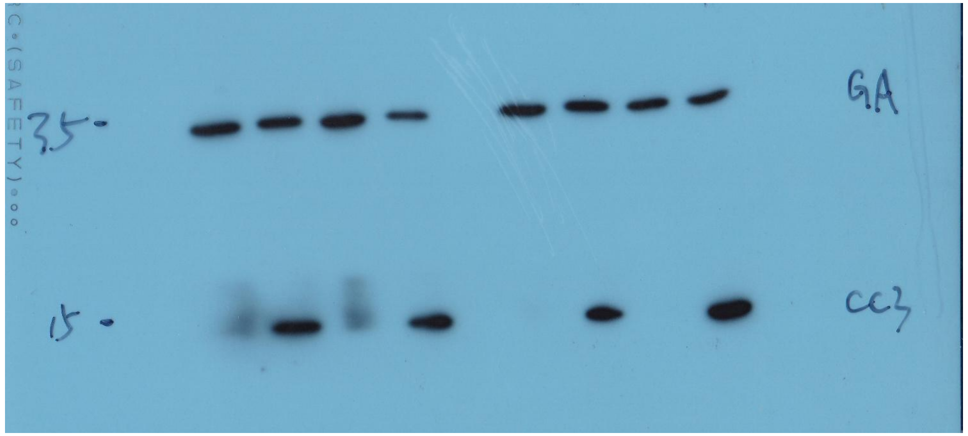

Supplement: Supplementary file 2 — Full and uncropped western blots [file 41420_2024_2099_MOESM2_ESM.pdf]
